# The Treatment of Tonsils and Adenoids

**Published:** 1921-12

**Authors:** John Kynaston

**Affiliations:** Lieut-Colonel R.A.M.C. (ret. pay)


### The Treatment of Tonsils and Adenoids.

*To the Editor of* The Hospital and Health Review.

Sir.—I note that you have an editorial in which you state that I wrote to "The Times" saying the operation of enlarged tonsils and adenoids was of no value, and that actual harm might be done by operating, and enlarged tonsils and adenoids might be left alone or treated by means other than operation. You insinuate that I am in favour of inaction, of allowing the children slowly to become diseased and deaf—you then quote what you describe as an admirable picture postcard, with the object of popularising the operation, which you commend to my attention.

Greater travesty upon what I said or upon the opinions which I hold could hardly be presented to your readers. Unfortunately, so many people read hurriedly and fail to grasp the plain meaning of the words used, and allow themselve to be hypnotised by their own preconceived ideas. My contention is that a vast number of operations are taking place all over the kingdom owing to a mistaken diagnosis of diseased tonsils and adenoids, not that an operation is unnecessary for the removal of these glands when they are as a fact diseased. Repeated observations show that only 5 per cent. of tonsils removed are diseased, then what justification is there for the removal of the other ninety-five? In cases of submerged tonsils, the removal of the plica triangularis is undoubtedly a very valuable proceeding in many cases, but why should the gland be removed when a circumcision is all that is necessary?

The symptoms which you quote with approval from the French postcard are obviously those of a nasal obstruction to a lesser or greater extent quite common after colds, influenza, measles, and whooping-cough. Am I to understand that you approve of removing the third tonsil because it is in an engorged condition? If not, why should you hold me up to ridicule for my contention that an operation under such conditions is unnecessary and unjustifiable, and that these symptoms are easily curable by medical means? It is no good denying that operations are done for these conditions, because the evidence is overwhelming. The truth is that there is a craze for such operations, and no attempt is made to differentiate between those that really require operations and those that do not.

It may be that I have discovered a peculiarly effective method of curing the nasal sepsis causing the obstruction, and, if so, surely medical practitioners should take the trouble to learn my methods, concerning which I make no secret, but to condemn or ridicule my practice in entire ignorance of its nature and results is not criticism, but merely prejudice incompatible with a scientific review of evidence.

Yours truly,
John Kynaston,
Lieut-Colonel R.A.M.C. (ret. pay).

[We print this letter from Dr. John Kynaston, although we do not necessarily agree with his inferences and conclusions.—Editor, H. & H.R.]

